# Periplosides Extract from *Cortex periplocae* Improve Collagen Antibody-Induced Arthritis by Regulating Macrophage Polarization

**DOI:** 10.3390/cimb46120843

**Published:** 2024-12-13

**Authors:** Que Wang, Xiaoyu Xiong, Li Chen, Fenghua Zhu, Xiaoqian Yang, Weimin Zhao, Shijun He, Jianping Zuo, Zemin Lin

**Affiliations:** 1 Experiment Center for Science and Technology, Shanghai University of Traditional Chinese Medicine, Shanghai 201203, China; 18616833881@163.com; 2 Laboratory of Immunopharmacology, State Key Laboratory of Drug Research, Shanghai Institute of Materia Medica, Chinese Academy of Sciences, Shanghai 201203, China; xiongxiaoyu@simm.ac.cn (X.X.); chenli@simm.ac.cn (L.C.); fhzhu@simm.ac.cn (F.Z.); xqyang@simm.ac.cn (X.Y.); 3 Department of Natural Product Chemistry, Shanghai Institute of Materia Medica, Chinese Academy of Sciences, Shanghai 201203, China; wmzhao@simm.ac.cn; 4 Innovation Research Institute of Traditional Chinese Medicine, Shanghai University of Traditional Chinese Medicine, Shanghai 201203, China

**Keywords:** rheumatoid arthritis, *Cortex periplocae*, pregnane glycosides, anti-arthritic activity, collagen antibody-induced arthritis, macrophage polarization

## Abstract

Rheumatoid arthritis (RA) is an inflammatory autoimmune disease characterized primarily by the synovial infiltration of inflammatory cells. Macrophage infiltration in the joint synovium is one of the early hallmarks of RA disease activity. *Cortex periplocae*, which has been widely employed in traditional Chinese medicine (TCM) to alleviate RA, harbors a bioactive compound known as *Periploca sepium* periplosides (PePs). In this study, collagen antibody-induced arthritis (CAIA) was established in mice through the administration of collagen antibodies and lipopolysaccharide (LPS), followed by treatment with PePs. The therapeutic effects of PePs were evaluated by measuring paw thickness, clinical arthritis scores, and histological changes in joint tissues. Flow cytometry and qRT-PCR were used to assess macrophage polarization in vivo and in vitro. The findings indicate that PePs effectively attenuated CAIA by suppressing the polarization of RAW264.7 cells towards the M1 phenotype while promoting their polarization towards the M2 phenotype. These results provide valuable insights into the scientific significance of PePs as a potential therapeutic agent for RA.

## 1. Introduction

Rheumatoid arthritis (RA) is an autoimmune disease characterized by tenosynovitis, leading to cartilage destruction and bone erosion, affecting multiple joints bilaterally [1]. RA significantly diminishes patients’ quality of life and imposes a substantial economic burden on both patients’ families and society. Severe complications, such as rheumatoid nodules, rheumatoid vasculitis, and cardiovascular diseases, which are also the primary causes of death, can arise in patients with RA [2]. Currently, the etiology of rheumatoid arthritis remains unclear. While immunosuppressive therapy based on autoimmune disorders can alleviate symptoms and delay the progression of rheumatoid arthritis, it cannot provide a cure [3].

Various natural products have been consistently studied for their potential in treating rheumatoid arthritis [4]. *Cortex periplocae*, also known as ‘Xiangjiapi’, is a traditional Chinese herbal medicine, derived from the root bark of *Periploca sepium* Bunge [5]. It has been extensively used as a folk medicine agent for treating rheumatoid arthritis in China [5,6]. As reported, pregnane glycosides, the primary components of *Cortex periplocae*, exhibit promising anti-inflammatory activity [7]. We prepared *Periploca sepium* periplosides (PePs) as a pregnane glycoside fraction free of cardiac glycosides, extracted from the root bark of *Cortex periplocae.* PePs consist of eight main pregnane glycosides: periplocosides A (PSA), C, D, E, K, O, Q, and R. In our team’s previous research, we discovered that PSA, which is a major component of PePs, exhibits superior immunosuppressive activity both in vitro and in vivo [8]. Furthermore, we established collagen-induced arthritis (CIA) and rat adjuvant-induced arthritis (AIA) models to evaluate the therapeutic efficacy of PePs, and the results demonstrated their remarkable anti-arthritis effects [6]. AIA and CIA are acknowledged to rely on T-cell activity, as they are induced through antigen-specific T-cell activation and migration [9]. Conversely, collagen antibody-induced arthritis (CAIA) is characterized by its independence from either T cells or B cells, as its induction is attributed to the infiltration of neutrophils and macrophages into the joints. In CAIA models, joint damage is initiated by targeting collagen type II, a key structural component of cartilage, through a cocktail of monoclonal antibodies. These antibodies bind to specific regions of the collagen, triggering an inflammatory response that leads to joint tissue degradation. To amplify this inflammatory cascade and accelerate joint swelling and damage, lipopolysaccharide (LPS) is administered after the antibody treatment. This combination of immune activation and inflammation contributes to the progressive destruction of the joint structure [10]. Hence, further investigation is warranted to fully explore the potential therapeutic effects of PePs on CAIA.

The utilization of the CAIA model in mice offers a direct and effective method for exploring the fundamental mechanisms implicated in rheumatoid arthritis pathogenesis, as well as facilitating the screening of potential therapeutic agents [11]. In light of our previous research findings, the primary objective of this study is to evaluate the therapeutic efficacy of PePs in the treatment of CAIA through the modulation of macrophage polarization in an in vivo setting, thus building upon our existing findings. Furthermore, we seek to simulate the process of polarization using RAW264.7 cells in vitro, aiming to investigate the specific impact of PePs on macrophage polarization.

## 2. Material and Methods

### 2.1. Plant Material and Extractions

The underground parts of *P. sepium* were collected and authenticated by Prof. Weimin Zhao from the Shanghai Institute of Materia Medica. Information about the preparation of PePs was described in our previous research [6].

### 2.2. Animals and Ethics Committee

Female BALB/c (age, 8–10 weeks old) were raised in specific pathogen-free (SPF) environments at the Animal Experimental Center of Shanghai Institute of Materia Medica. Standard laboratory food and water were disinfected and freely accessible to all the mice. The mice were housed in a temperature-regulated environment at 22 °C and 50% humidity with a 12 h light/dark cycle.

The animal experiments were conducted in a manner that is consistent with ethical principles and approved by the Institute Animal Care and Use Committee (IACUC) at Shanghai Institute of Materia Medica, Chinese Academy of Science, with IACUC approval number 2020-06-ZJP-121.

### 2.3. Induction and Treatment of CAIA Mice

Mice were randomly divided into six groups based on body weight: normal group, CAIA group, CAIA + Dex group (1 mg/kg dexamethasone), CAIA + PePs (12.5 mg/kg) group, CAIA + PePs (6.25 mg/kg) group, and CAIA + PePs (3.13 mg/kg) group. Except for the normal group, arthritis mice were intraperitoneally injected with a mixture of type II collagen monoclonal antibodies (1.5 mg/mouse, Woodinville) and then injected with lipopolysaccharide (LPS, 15 μg/mouse) into the peritoneal cavity three days later. The normal group of mice received an equal amount of phosphate-buffered saline (PBS). Two days later, the CAIA mice were orally administered saline, and the CAIA + Dex group received a daily oral gavage of dexamethasone (1 mg/kg), while the three PePs groups were administered a daily oral gavage of PePs at the indicated doses (12.5 mg/kg, 6.25 mg/kg, 3.13 mg/kg).

### 2.4. Assessment of Arthritis

As previously reported, the thickness of the hind paws of each mouse was measured using a micrometer, and clinical arthritis scores were evaluated daily using a blind approach [12].

### 2.5. Histopathological Examination

The hind joint tissues of the mice were soaked in 10% formalin for 48 h at the end of the experiment. After fixation, the joint specimens were rinsed with phosphate-buffered saline and immersed in 5% formic acid decalcifying solution. After dehydration with an ethanol gradient of different concentrations, the decalcified tissue was made transparent using xylene, followed by embedding and slicing.

The sectioning of tissues (5 μm) was performed with Hematoxylin–Eosin (H&E) staining, safranin O-fast green (OF) staining, and toluidine blue (TB) staining, to observe the damage of synovial tissue, cartilage tissue, and bone tissue, respectively. The sections were examined under a microscope with 50× magnification.

### 2.6. In Vitro Polarization of Macrophages and Treatments

Mouse macrophage RAW264.7 cell lines (ATCC) were cultured in DMEM (Gibco, Waltham, MA, USA) containing 10% fetal bovine serum (FBS, Hyclone, Logan, UT, USA) and antibiotics of 100 U/mL penicillin and 100 µg/mL streptomycin (North China Pharmaceutical, Shijiazhuang, China). Cell culturing was carried out in an incubator containing 5% CO_2_ and maintained at 37 °C.

The experimental setup aimed to simulate macrophage polarization from the M0 state to M1 (pro-inflammatory) or M2 (anti-inflammatory) phenotypes. RAW264.7 macrophages were preincubated with PePs at different concentrations (1 μg/mL, 0.5 μg/mL, 0.25 μg/mL) for 1 h. Five groups were established: a blank control group without IFN-γ/LPS or IL-4 stimulation; a control group with IFN-γ/LPS (for M1 polarization) or IL-4 (for M2 polarization) stimulation; and three groups pretreated with different concentrations of PePs followed by IFN-γ/LPS or IL-4 stimulation. RAW264.7 cells were preincubated with PePs at different concentrations of 1 μg/mL, 0.5 μg/mL, and 0.25 μg/mL for 1 h. After 1 h, the cells were stimulated with 2.5 ng/mL of recombinant mouse IFN-γ (BD Bioscience) and 200 ng/mL LPS to promote the differentiation of RAW264.7 to M1 macrophages or 10 ng/mL IL-4 (BD Bioscience) for the RAW264.7 cells differentiating to M2 macrophages in the absence or presence of PePs at indicated concentrations for 6 h, except for the blank control group. For flow cytometry analysis, Protein Transport Inhibitor (BD Bioscience) was added at a dose of 1 µL/well simultaneously with the addition of IL-4.

After 6 h of treatment, the cells were analyzed by flow cytometry or RT-qPCR to assess macrophage polarization.

### 2.7. Flow Cytometric Analysis

The execution of mice and the preparation of spleen lymphocytes or RAW264.7 were conducted. In the flow cytometry analysis, spleen lymphocytes or RAW264.7 macrophages were blocked using anti-mouse CD16/CD32 to prevent non-specific Fc receptor binding. The cells were then stained with the following antibodies: PE-conjugated CD45, PerCP-Cy5.5-conjugated CD11b, FITC-conjugated F4/80, Alexa Fluor^®^ 647-conjugated CD206, and PE-Cyanine7-conjugated iNOS. Macrophages were defined as CD45^+^CD11b^+^F4/80^+^, with M1 macrophages identified as CD11b^+^F4/80^+^iNOS^+^ and M2 macrophages as CD11b^+^F4/80^+^CD206^+^ or CD11b^+^F4/80^+^IL-10^+^.

Flow cytometry was performed using BD LSRFortessa™ (BD Biosciences, Franklin Lakes, NJ, USA), and data analysis was conducted with FlowJo software (v10.9.0).

### 2.8. Quantitative Real-Time Polymerase Chain Reaction (RT-qPCR)

The extracted RNA was reverse-transcribed using Hifair II 1st Strand cDNA Synthesis Super-Mix for qPCR (gDNA digester plus, Yeasen, Shanghai, China) and quantified using nanodrop. A real-time PCR was performed using Hieff qPCR SYBR Green Master Mix (High Rox, Yeasen) on an Applied Biosystems 7900HT Fast Real-Time PCR System. RT-qPCR was conducted to assess the expression levels of several pro-inflammatory markers associated with M1 macrophages (such as iNOS, COX-2, IL-1β, MMP2, and MMP9) and anti-inflammatory markers associated with M2 macrophages (such as Ym-1, Arg-1, and Fizz-1). The primers used for PCR amplification are listed in Supplementary Table S1.

### 2.9. Statistical Analysis

GraphPad Prism was used for data analysis. Statistical analysis was performed using GraphPad Prism 9.4.1 (GraphPad Software Inc., San Diego, CA, USA). A one-way ANOVA was used for experiments with multiple groups, and a two-way ANOVA was used for measuring arthritis scores and mouse paw thickness, both of which were followed up by Dunnett’s multiple comparison test. Data with a *p* value less than 0.05 were considered significant.

## 3. Results

### 3.1. PePs Treatment Alleviated CAIA in Mice

While our recent study demonstrated that the efficacy of PePs in reducing joint swelling and mitigating bone erosion in AIA and CIA models depended on T-cell activity; there remains a knowledge gap regarding its specific effects on disease activity in the CAIA model which is characterized by its independence from both T cells and B cells [9,10]. Hence, further investigation was conducted to fully explore the potential therapeutic effects of PePs on CAIA.

**Figure 1:**
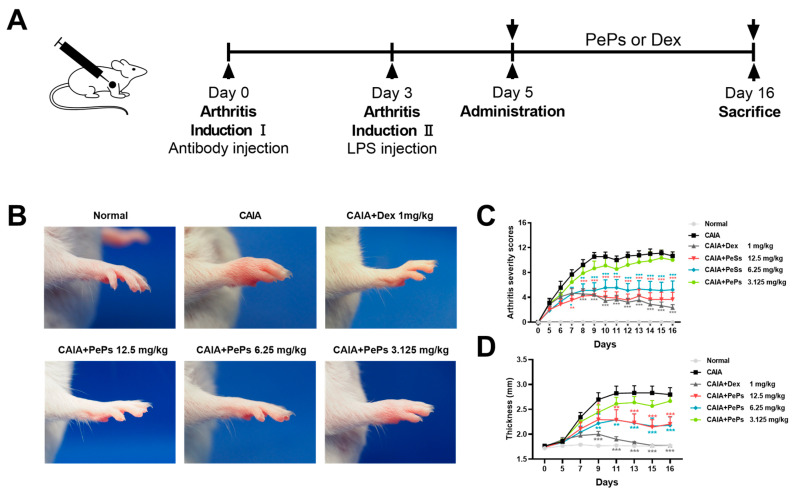
Anti-arthritis effects of PePs in mouse CAIA model. (**A**) Timeline of CAIA induction and administration of PePs in BALB/c mice. Oral administration of PePs (12.5 mg/kg, 6.25 mg/kg, and 3.125 mg/kg) and Dex (1 mg/kg) was performed once daily. Representative diagram of paw morphology (**B**). Arthritis severity scores (**C**) and the thickness (**D**) were assessed daily in blinded manner. Data are presented as mean ± SD. * *p* < 0.05, ** *p* < 0.01, *** *p* < 0.005 significant as compared to vehicle group, *n* = 9.

The CAIA mice received an oral administration of saline, PePs (12.5 mg/kg, 6.25 mg/kg and 3.125 mg/kg), or dexamethasone (1 mg/kg) once daily for consecutive days (Figure 1A). As depicted in Figure 1B, representative photographs demonstrate the reversal of arthritic severity in CAIA mice, including erythema and swelling, upon treatment with both dexamethasone (Dex) and PePs. PePs exhibited a dose-dependent inhibition of inflammation in CAIA mice, as indicated by the clinical assessments of arthritis and the measurements of mouse paw thickness (Figure 1C,D).

### 3.2. PePs Treatment Attenuated Histological Symptoms of CAIA in Mice

**Figure 2:**
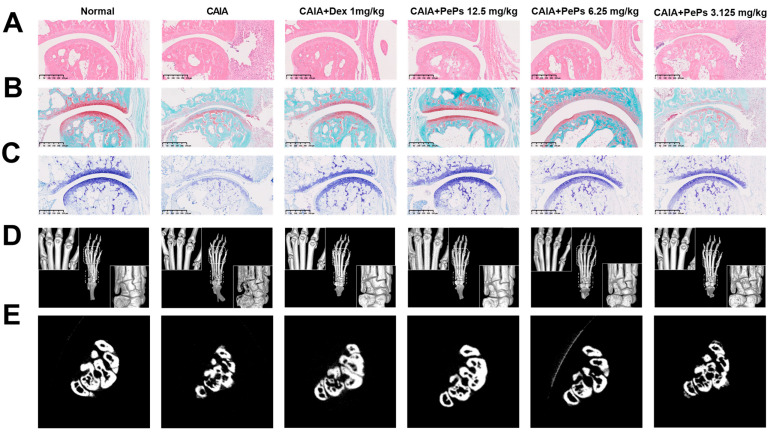
Anti-arthritis effects of histological symptoms by PePs in mouse CAIA model. Representative images of ankle joint sections stained with H&E (**A**), toluidine blue (**B**), and safranin O-fast green (**C**). Representative 3D reconstruction (**D**) and cross-section (**E**) images of right hind paws of mice from each group.

Notably, PePs significantly ameliorated the local injection of type II collagen monoclonal antibody and LPS-induced immune cell infiltration, joint cartilage loss, the exposure of subchondral bone, and structural damage (Figure 2A–C). Dex, the positive control, a widely prescribed corticosteroid, also demonstrated superior resistance against pathological changes in CAIA mice. Additionally, bone mineral density, evaluated using Micro-CT, revealed that compared to the control group, CAIA mice exhibited severe bone erosion, while mice treated with Dex or PePs (at doses of 12.5 mg/kg and 6.25 mg/kg) displayed reduced bone resorption (Figure 2D,E). These findings collectively suggest that PePs effectively mitigate joint inflammation, cartilage destruction, and bone erosion in CAIA mice.

### 3.3. PePs Regulated the Polarization of Macrophages In Vivo

**Figure 3:**
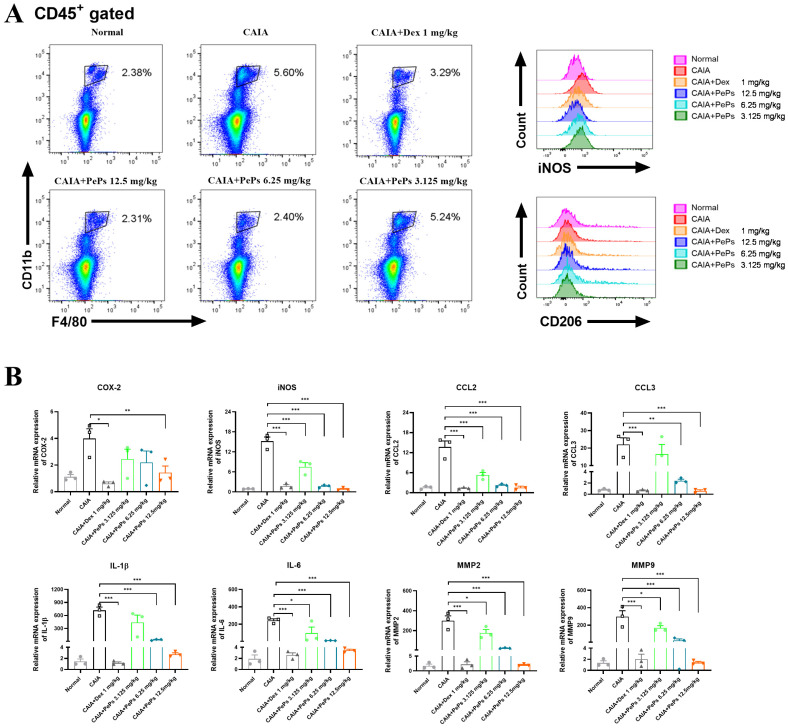
PePs regulate polarization of M1 and M2 in vivo. Representative flow cytometry analysis (**A**) of splenic macrophages in CAIA mice gated by CD45^+^, and intracellular expression of iNOS and CD206 in each macrophage population, represented as flow histograms. mRNA levels of COX-2, iNOS, CCL2, CCL3, IL-1β, IL-6, MMP2, and MMP9 in mice from each group measured by qRT-PCR (**B**). Data are presented as mean ± SEM. * *p* < 0.05, ** *p* < 0.01, *** *p* < 0.001, indicating significance compared to vehicle/CAIA group.

The infiltration of M1 macrophages into the synovium and the subsequent increase in the M1/M2 ratio are considered indicators of rheumatoid arthritis progression. Flow cytometric analysis further demonstrated that PePs regulated macrophage polarization in vivo. Specifically, the frequency of CD11b^+^F4/80^+^ macrophages expressing iNOS (M1 marker) was reduced in splenocytes, indicating a shift from pro-inflammatory M1 macrophages to anti-inflammatory M2 macrophages (CD11b^+^F4/80^+^CD206^+^, M2 marker) (Figure 3A). These findings collectively suggest that PePs alleviate joint inflammation and structural damage in CAIA mice while promoting a shift toward anti-inflammatory macrophage phenotypes. At the gene expression level, PePs treatment resulted in attenuated mRNA expression levels of pro-inflammatory factors, including COX-2, iNOS, CCL2, CCL3, IL-1β, IL-6, MMP2, and MMP9, secreted by M1 macrophages at the joint site in CAIA mice (Figure 3B). The in vivo results indicate that PePs have the potential to ameliorate CAIA by modulating macrophage polarization.

### 3.4. PePs Regulated the Polarization of Macrophages In Vitro

**Figure 4:**
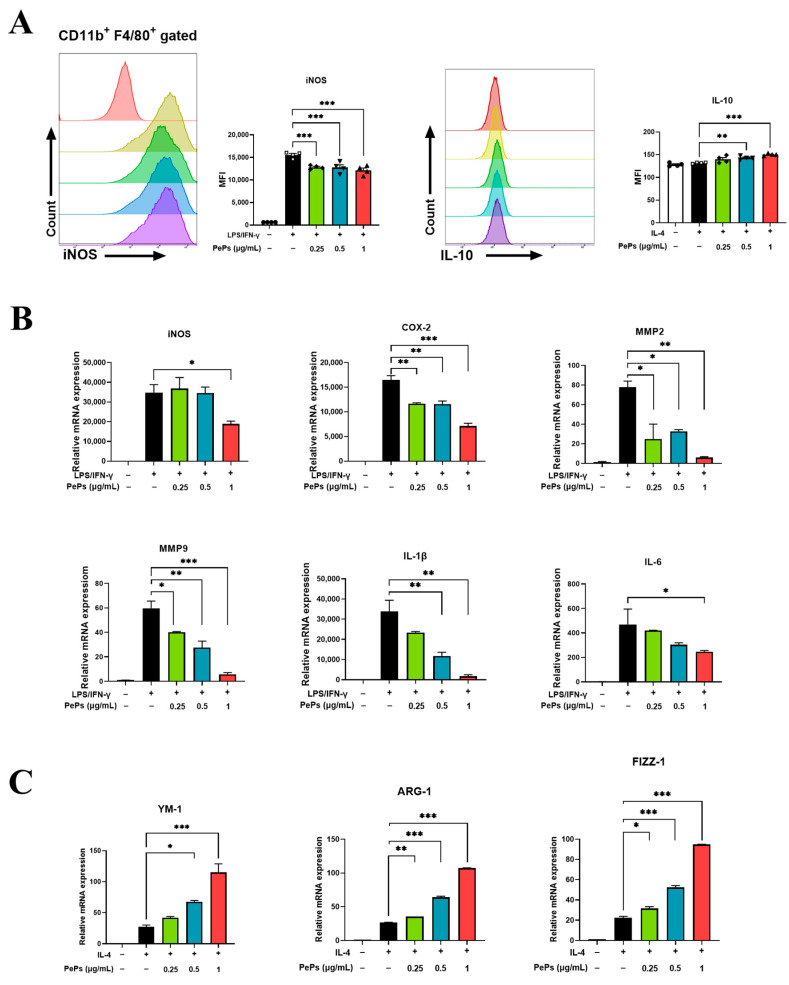
PePs regulate polarization of M1 and M2 on RAW264.7 cells. Representative flow cytometry histograms and mean fluorescence intensity of iNOS^+^ cells or CD206^+^ cells among macrophage populations (gated by CD11b^+^F4/80^+^) (**A**). mRNA levels of COX-2, iNOS, IL-1β, IL-6, MMP2, and MMP9 in RAW264.7 cells stimulated with LPS/IFN-γ with or without preincubation of indicated concentrations of PePs for 6 h, assessed by qRT-PCR (**B**). mRNA levels of YM-1, ARG-1, and FIZZ-1 in RAW264.7 cells stimulated with IL-4, with or without preincubation of indicated concentrations of PePs for 6 h, assessed by qRT-PCR. (**C**) Data are presented as mean ± SEM. * *p* < 0.05, ** *p* < 0.01, *** *p* < 0.001, indicating significance compared to LPS/IFN-γ or IL-4 group.

Flow cytometry analysis further revealed that PePs decreased the expression of iNOS, a marker of M1 macrophages, and increased the expression of the anti-inflammatory factor IL-10 secreted by M2 macrophages (Figure 4A). In vitro cell simulations using RAW264.7 cells stimulated with LPS/IFN-γ to induce M1 polarization yielded results consistent with those obtained from the animal models (Figure 4B and Supplementary Figure S1). Additionally, at the cellular level, PePs increased the mRNA expression of Ym-1, Arg-1, and Fizz-1, which serve as markers for M2 macrophages (Figure 4C). Collectively, our results demonstrate that PePs can inhibit the polarization of M0 macrophages toward the M1 phenotype while promoting their polarization toward the M2 phenotype, both in vitro and in vivo.

## 4. Discussion

One of the prominent pathological features of RA is the local infiltration of immune cells, particularly the abundance of macrophages in the synovial tissue site [13]. Macrophages can polarize towards M1 (classical activation) or M2 (alternative activation) in response to local microenvironmental stimuli. Imbalance in the M1/M2 macrophage ratio, with a predominance of M1 macrophages, has been implicated in the formation of osteoclasts in RA patients, leading to persistent inflammation and bone erosion [14,15,16].

In recent years, there has been a growing body of research dedicated to exploring the pathophysiological implications of the innate immune system in the context of RA. In RA patients, augmented expression levels of pro-inflammatory molecules serve as stimuli that instigate the mobilization of monocytes, particularly those within the intermediate subset, directing them towards the synovial tissue where they subsequently undergo differentiation into M1 macrophages [17]. This phenomenon is notably characterized by an enhanced prevalence of activated pro-inflammatory macrophages within the synovial tissue, which is recognized as an early distinguishing feature of RA and is positively associated with a higher ratio of M1 macrophages in comparison to M2 macrophages [17,18]. Moreover, Cheng et al. demonstrated that inhibitors of peptidyl arginine deiminase-4 can alleviate arthritis symptoms by reducing M1 polarization [19].

In this study, we demonstrated that PePs significantly improved CAIA by modulating macrophage polarization. PePs treatment decreased M1 macrophage markers, including iNOS, COX-2, IL-1β, and IL-6, while enhancing M2 markers such as Arg-1, Ym-1, and Fizz-1. Flow cytometry analysis indicated that PePs inhibited the formation of iNOS^+^ M1 macrophages and promoted the increase in CD206^+^ M2 macrophages both in vivo and in vitro. While our previous work demonstrated the anti-inflammatory properties of PePs, the current study provides a more comprehensive understanding of their mechanism of action by directly linking macrophage polarization to the attenuation of CAIA symptoms. Importantly, these results suggest that PePs may offer therapeutic potential for RA by modulating the innate immune response and restoring macrophage homeostasis. This finding aligns with recent evidence showing that naturally derived bioactive compounds can modulate innate immune responses, similar to the way vitamin D influences macrophage activity by promoting anti-inflammatory states through pathways like TLR2 activation. These properties suggest that PePs may act in a complementary manner to known immunomodulators, such as vitamin D, in regulating inflammatory pathways in RA, thereby offering potential therapeutic benefits [20].

The pregnane glycoside structure of PePs, similar to other bioactive pregnane compounds, is known to influence macrophage activity and inflammation. Structurally related pregnane glycosides from other species have shown the potent inhibition of pro-inflammatory mediators, such as nitric oxide, TNF-α, and IL-1β, by targeting macrophages and inhibiting signaling pathways associated with inflammation [21]. These findings suggest that the pregnane glycoside backbone, with its specific glycosidic linkages, may contribute to PePs’ ability to reduce the M1/M2 macrophage ratio, thus restoring immune balance and reducing inflammation in RA models. Our results align with these findings, suggesting that the structural properties of PePs contribute to their broader immunomodulatory effects, including the regulation of macrophage activity.

**Figure 5:**
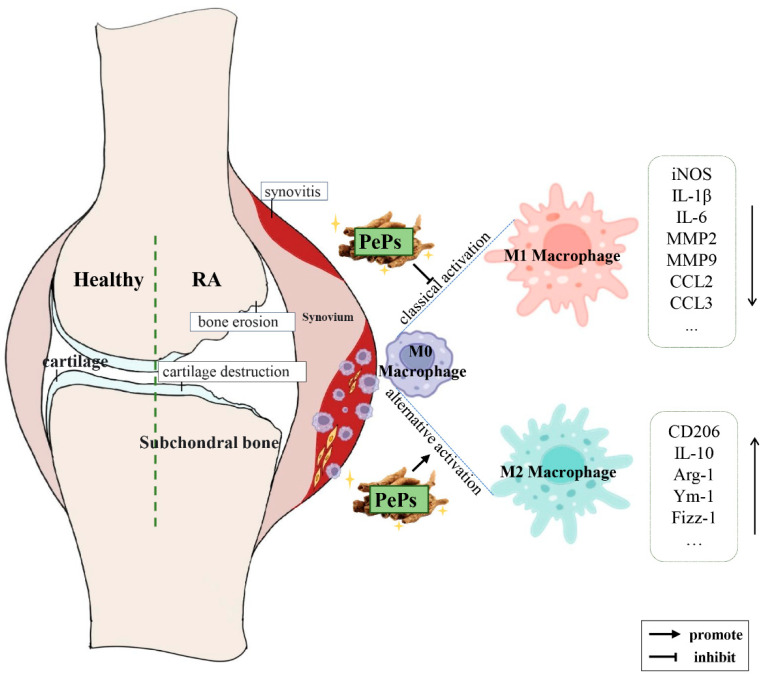
Schematic diagram of underlying therapeutic mechanisms of PePs. PePs relieve arthritis by modulating macrophage polarization between M1 and M2 phenotypes. Downward arrows indicate a decrease in the expression of relevant markers, while upward arrows indicate an increase in the expression of relevant markers.

In summary, our findings demonstrate that PePs exert anti-inflammatory and anti-arthritic effects in vivo in a dose-dependent manner. Moreover, we investigated their potential mechanism of action in modulating macrophage polarization as an anti-arthritic strategy (Figure 5). Based on our results, we suggest that PePs could be a potential alternative therapy for RA. Therefore, further studies are warranted to elucidate the intracellular signaling pathways involved in the PePs-mediated regulation of macrophage polarization and to evaluate the possible side effects of PePs as an antirheumatic agent.

## Data Availability

The data that support the findings of the present study are available from the corresponding author on reasonable request.

## Supplementary Materials

The following supporting information can be downloaded at: https://www.mdpi.com/article/10.3390/cimb46120843/s1.

## Abbreviations

AIA	adjuvant-induced arthritis
CAIA	collagen antibody-induced arthritis
CIA	collagen-induced arthritis
ConA	concanavalin A
DEX	dexamethasone
DMARD	disease-modifying antirheumatic drug
DNFB	2,4-dinitro-1-fluorobenzene
EAE	experimental autoimmune encephalomyelitis
H&E staining	Hematoxylin–Eosin staining
IACUC	Institute Animal Care and Use Committee
LPS	lipopolysaccharide
MFI	mean fluorescence intensity
NC	normal control
OF staining	safranin O-fast green staining
PBS	phosphate-buffered saline
PePs	periploca sepium periplosides
PSA	periplocoside A
PSE	periplocoside E
qRT-PCR	quantitative real-time polymerase chain reaction
RA	rheumatoid arthritis
SPF	specific pathogen-free
TB staining	toluidine blue staining
TCM	traditional Chinese medicine
