# Supplementary material for: Periplosides Extract from Cortex periplocae Improve Collagen Antibody-Induced Arthritis by Regulating Macrophage Polarization
Source: Curr Issues Mol Biol. 2024 Dec 13;46(12):14095–105. doi: 10.3390/cimb46120843 (PMC11674450; doi:10.3390/cimb46120843)
Supplement: Supplementary file 1 [file cimb-46-00843-s001.zip › cimb-3290581-supplementary.pdf]

Supplementary Materials and Methods

Title: Periplosides extract from *Cortex periplocae* improve collagen antibody-induced arthritis by regulating macrophage polarization

Authors: Que Wang [1,†], Xiaoyu Xiong [2,†], Li Chen [2], Fenghua Zhu [2], Xiaoqian Yang [2], Weimin Zhao [3], Shijun He [4], Jianping Zuo [1,2,*] and Zemin Lin [1,2,*]

† These authors contributed equally to this work.

* Corresponding Authors.

TABLE S1.

Names and sequences of primers used for polymerase chain reaction analysis.

Gene	Sequence
iNOS	F: 5’-CAGCTGGGCTGTACAAACCTT-3’<br>R: 5’-CATTGGAAGTGAAGCGTTTCG-3’
COX-2	F: 5’-CAGCAAAGCCTAGAGCAACAA-3’<br>R: 5’-CGGAACTAAGAGGAGCAGCAAT-3’
CCL2	F: 5’-TTAAAAACCTGGATCGGAACCAA-3’<br>R: 5’-GCATTAGCTTCAGATTTACGGGT-3’
CCL3	F: 5’-TGCGCTGACTCCAAAGAGAC-3’<br>R: 5’-CTCGATGTGGCTACTTGGCA-3’
IL-1β	F: 5’-AGTTGACGGACCCCAAAAG-3’<br>R: 5’-TTTGAAGCTGGATGCTCTCAT-3’
IL-6	F: 5’-CTCCGACTTGTGAAGTGGTATAG-3’<br>R: 5’-CCACCTCAATGGACAGAATATCA-3’
MMP2	F: 5’-AGTTTGGTGTCGCGGAGCAC-3’<br>R: 5’-TACATGAGCGCTTCCGGCAC-3’
MMP9	F: 5’-AGTTTGGTGTCGCGGAGCAC-3’<br>R: 5’-TACATGAGCGCTTCCGGCAC-3’
YM-1	F: 5’-CAGGTCTGGCAATTCTTCTGAA-3’<br>R: 5’-GTCTTGCTCATGTGTGTAAGTGA-3’
ARG-1	F: 5’-CTCCAAGCCAAAGTCCTTAGAG-3’<br>R: 5’-AGGAGCTGTCATTAGGGACATC-3’
FIZZ-1	F: 5’-CCAATCCAGCTAACTATCCCTCC-3’<br>R: 5’-CCAGTCAACGAGTAAGCACAG-3’
β-actin	F: 5’-CCACCATGTACCCAGGCATT-3’

R: 5'-AGGGTGTAAAACGCAGCTCA-3'

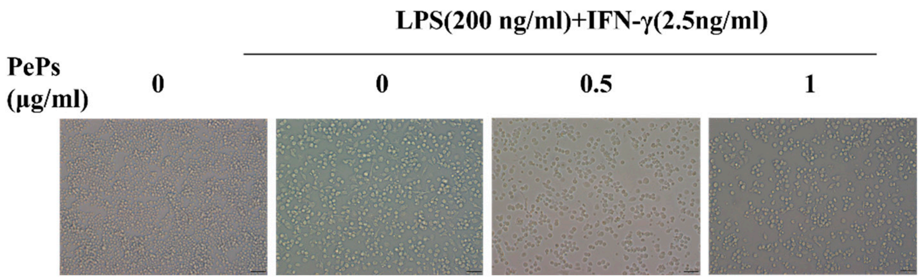

FIGURE S1.

PePs inhibited the polarization of macrophages.

RAW264.7 cells were stimulated with IFN-γ and LPS to promote the differentiation of RAW264.7 to M1 macrophages. Cell morphology was observed under a microscope.
